# Characterization of Biodegraded Ignitable Liquids by Headspace–Ion Mobility Spectrometry

**DOI:** 10.3390/s20216005

**Published:** 2020-10-23

**Authors:** José Luis P. Calle, Marta Ferreiro-González, María José Aliaño-González, Gerardo F. Barbero, Miguel Palma

**Affiliations:** Department of Analytical Chemistry, Faculty of Sciences, Agrifood Campus of International Excellence (ceiA3), IVAGRO, University of Cadiz, 11510 Puerto Real, Spain; joseluis.perezcalle@uca.es (J.L.P.C.); mariajose.alianogonzalez@alum.uca.es (M.J.A.-G.); gerardo.fernandez@uca.es (G.F.B.); miguel.palma@uca.es (M.P.)

**Keywords:** ignitable liquids, biodegradation, sensor, characterization, headspace, ion mobility spectrometry, chemometrics

## Abstract

The detection of ignitable liquids (ILs) can be crucial when it comes to determining arson cases. Such identification of ILs is a challenging task that may be affected by a number of factors. Microbial degradation is considered one of three major processes that can alter the composition of IL residues. Since biodegradation is a time related phenomenon, it should be studied at different stages of development. This article presents a method based on ion mobility spectroscopy (IMS) which has been used as an electronic nose. In particular, ion mobility sum spectrum (IMSS) in combination with chemometric techniques (hierarchical cluster analysis (HCA) and linear discriminant analysis (LDA)) has been applied for the characterization of different biodegraded ILs. This method intends to use IMSS to identify a range of ILs regardless of their degree of biodegradation. Three ILs (diesel, gasoline and kerosene) from three different commercial brands were evaluated after remaining in a soil substrate for several lengths of time (0, 2, 5, 13 and 38 days). The HCA results showed the samples’ trend to fall into categories characterized by ILs type and biodegradation time. The LDAs allowed a 99% successful classification of the samples according to the IL type. This is the first time that an HS-IMS technique has been used to detect ILs that have undergone biodegradation processes. The results show that IMS may be a promising alternative to the current standard method based on gas-chromatography for the analysis of biodegraded ILs. Furthermore, no pretreatment of the samples nor the use of a solvent is required.

## 1. Introduction

Forest fires are one of the major environmental problems in our days, since they represent significant economic losses and, in the worst of scenarios, fatal casualties [1,2]. Most forest fires are caused by human activity (either intentionally or accidentally) where ignitable liquids (ILs) usually play an important role. The most often found IL in fire debris is gasoline because of its low cost and ready availability [3]. Detecting ILs is a major concern in fire investigations. However, many factors make of this task an extremely difficult one. Some of those factors are: the inherent destructive nature of fire, the intervention of firefighters using huge amounts of water or fire suppression agents, the low trace level of the ILs that usually remain at fire scenes, etc. [4,5]. In addition, fire debris sampling is carried out several hours or even days after the fire has been extinguished and, in some cases, they are stored for extended periods of time prior to their analysis. During that time, the natural phenomenon of degradation by evaporation as well as by microbial activity (biodegradation) may affect ILs’ chemical composition, which would naturally hinder their proper identification [6,7].

Different analytical techniques have been described for the correct identification and classification of ILs [8]. So far, the method par excellence currently used by most forensic laboratories was established by the American Society for Testing and Materials (ASTM) and is based on gas chromatography–mass spectrometry (GC-MS) [9]. Prior to their analysis by GC-MS, a pre-concentration step is required to separate and/or isolate those analytes of interest from the rest of the sample. For this purpose, the ASTM proposes different techniques such as solid-phase microextraction [10], tenax [11] or activated charcoal strips (ACS) [12]. However, these techniques present a number of disadvantages, since the use of fiber adsorbents increases the cost and time of the analysis, and also affect its useful life and robustness, among others. Moreover, some of these techniques, such as ACS, require the use of solvents such as carbon disulfide for the desorption of the strips. Carbon disulfide is a toxic substance and with a very low autoignition temperature, which poses an additional hazard to the analysis procedures. Additionally, Table 1 shows the main properties of these techniques.

Additionally, according to ASTM E1618 [9] ILs are classified into their corresponding category by identifying some of the individual compounds that are characteristically found in each category. Such classification is based on a visual comparison with a reference sample that intends to identify the total ion chromatogram (TIC) of the analyzed sample. For this purpose, an extensive online globally available reference database is provided by the National Center for Forensic Science [13]. Visual pattern recognition can be time consuming, since it does not allow automated database searching and it is a subjective methodology that depends, to some extent, on each analyst’s particular experience. This method has demonstrated its effectiveness when dealing with neat IL, however, in some cases, the samples may have undergone different phenomena that would affect their chromatographic profile and, in those case, the correct classification of the samples based on the visual comparison of their TICs may become rather difficult [13]. Based on the complex mixture of organic compounds that characterize ILs, which are mostly petroleum based-products, microbial degradation is one of the phenomena that should always be taken into consideration. In fact, the biodegradation is considered one of three major processes that can alter the composition of IL residues since bacteria use the IL as a carbon source [7]. For this reason, microorganisms are a challenge for fire debris analysts. Previous research studies have demonstrated that biodegradation leads to the loss of specific chemical compounds, this would imply a modification of proportions as well as of each IL’s general profile [6,7,14,15,16,17]. The degree of degradation depends on a number of factors [7] such as the type of microorganisms [18], the type of soil [19] and the length of time that such microorganisms are in contact with the IL. Thus, this biodegradation phenomenon is aggravated in many cases by sampling and/or analysis delay, hence the necessity to further study biodegradation as a time-dependent function [16]. Most studies until present have used potting soil as the substrate, since it contains a large variety of microorganisms [16,19].

The use of summed-ion mass spectrum TIS (total ion spectrum) together with chemometric techniques has been proposed as an effective alternative to the visual comparison of TICs [20]. TIS is the spectrum resulting from adding the intensity from each mass/charge to all the chromatographic times. The information related to the separation in the GC is discarded. In this way, global profiles, instead of individual compounds, are used for identification purposes. This approach facilitates an automated database searching as well as the identification of ILs in a rapid, and objective way. In fact TIS has been used for the identification of ILs in combination with different chemometric tools, including non-supervised techniques such as hierarchical cluster analysis (HCA) [21] as well as supervised techniques, such as linear and quadratic discriminant analysis (LDA or QDA) [7]).

With the introduction of TIS, non-separative techniques such as headspace mass spectrometry electronic nose (HS-MS eNose) became an alternative in this field [22,23]. This technique provides total ion mass spectrum (TIMS) in a similar way to TIS. However, by avoiding the chromatographic separation and the use of adsorbents, this alternative technique becomes cleaner, faster and more economic than the rest. The use of TIMS in combination with chemometric tools also allows its automated database searching and the results do not depend on the analyst’s previous experience. HS-MS eNose has been successfully applied and validated for the identification of neat ILs [23] as well as IL residues in fire debris [22]. This technique has also been used to determine the effect of weathering by evaporation on gasoline, diesel oil and paraffin [24,25]. The results obtained have demonstrated that the discrimination of different weathered ILs was successfully completed when TIMS was used in combination with LDA. However, the phenomenon of biodegradation with this technique has not been studied yet.

Based on this satisfactory use of TIMS, a recent study has demonstrated the potential of a novel method for the detection of ILs in fire debris by performing a direct analysis of the headspace by ion mobility spectrometry (IMS) instead of MS detector [26]. In this case, IMSS together with pattern recognition techniques, mainly HCA and LDA, was used for discrimination purposes. The IMSS is the result of the sum of the intensities at each drift time, in which each drift time acts as a “sensor”. Both HS-IMS and HS-MS eNose have advantages over conventional techniques such as short time requirements, no pretreatment of the sample and low detection limits (without the need for extraction and pre-concentration techniques). In addition, HS-IMS presents a greater portability, since it works at atmospheric pressure. This technique has been successfully used for the discrimination of samples in many fields including the detection of adulterated food [27,28], explosives [29,30], drugs or pharmaceuticals substances [31,32,33] as well as for the characterization of ILs in fire debris [26]. However, how biological degradation may affect the correct identification of ILs when this technique is used is still to be studied.

Therefore, our present work intends to determine the suitability of this technique to characterize different ILs in soil samples when they have been subjected to a biodegradation process for different periods of time (from hours to more than one month).

## 2. Materials and Methods

### 2.1. Samples

Three types of ILs (diesel, gasoline and kerosene) from different vendors in Spain were selected. From each IL type, three different brands were chosen in order to increase diversity, so that a total of nine ILs were finally analyzed. The soil commercialized under the name of “Fertilizer Blue Universal Novatec” by Compo (Münster, Germany) was used as the substrate for all the samples.

Biodegradation phenomena were simulated in a similar way presented in the literature [7,16]. 5 µL were added to approximately 1 g of potting soil and these samples were kept in 10 mL glass vials (Agilent Crosslab, Santa Clara, CA, USA). The vials were sealed (to avoid the weathering process) and stored under controlled temperature (25 °C). The degradation times for all the ILs samples ranged from 0 (approximately 1 h), 2, 5, 13 up to 38 days. A soil control sample consisting on 1 g of soil without any IL was also elaborated and kept under the same conditions and for the same length of time as the analysis samples. The samples were labeled as follows: the IL code (Gas for gasoline, Dies for diesel oil and Ker for kerosene) followed by the brand identification number (1,2,3), then, the degradation time (number of days) followed by R1 or R2 to indicate the replica number. The control samples free from ILs were identified as IL-free sample. A total of 100 samples (*n* = 100) were analyzed, 10 samples of IL-free and 90 samples containing ILs (3 ILs × 3 commercial brands × 5 different times × 2 replicates).

### 2.2. HS-GC-IMS Analysis Acquisition

All the samples were analyzed using a FlavourSpec system (G.A.S., Dortmund, Germany) which consists of an automatic headspace injector, a short GC column and an IMS detector. The samples stored in 10 mL sealed vials were placed in the autosampler oven to be heated and agitated in order to generate the headspace. The headspace (250 µL) was taken from the vials by means of a gas syringe and injected into an isotherm GC multicapillary column MCC OV-5 of 20 cm long (G.A.S., Dortmund, Germany) for the separation of the VOCs before they reached the IMS detector. The conditions for analysis had been previously optimized by our research team and can be seen in Table 2. The ionization method used was 3H Tritium beta radiation. Nitrogen (99.999% purity) was used as both, drift and carrier gas. In order to avoid cross-contamination, the gas syringe was flushed with carrier gas (nitrogen) after every sample injection.

### 2.3. Data Analysis

The data were acquired in positive mode by means of the software application LAV HS-GC-IMS (G.A.S., Dortmund, Germany). The ion mobility sum spectrum was obtained by adding up the intensities across the chromatographic profile according to the method described by Aliaño-González et al. [26,28]. In the resulting spectrum (intensity vs. drift times), each drift time acts as a “sensor”. The resulting IMSS included 990 drift times ranging from 1.020 to 2.010 (relative to the RIP (reaction ion peak)). Drift times are referenced to the RIP, which is the characteristic signal of the water molecules ionized by the source. The RIP is used as the internal standard since it provides a sharp signal at a specific position related to the cleanliness of the system. The normalization by plotting all the drift times relative to the RIP allows to minimize the equipment variations. Once the IMSS was obtained for the whole set of samples (*n* = 100) the spectra were normalized by assigning one unit to each sample’s maximum intensity. In this way, a final data matrix (D_mxn_), where *m* is the number of samples and *n* is the number of variables (drift times), was obtained.

The multivariate analysis of the data included a non-supervised pattern recognition method, such as hierarchical cluster analysis (HCA), which was performed by means of RStudio software (RStudio.2019, Boston, MA, USA). The supervised analysis, namely linear discriminant analysis (LDA) was performed by means of IBM SPSS Statistics 22 statistical software (SPSS Inc., Chicago, IL, USA).

## 3. Results and Discussion

**Figure 1:**
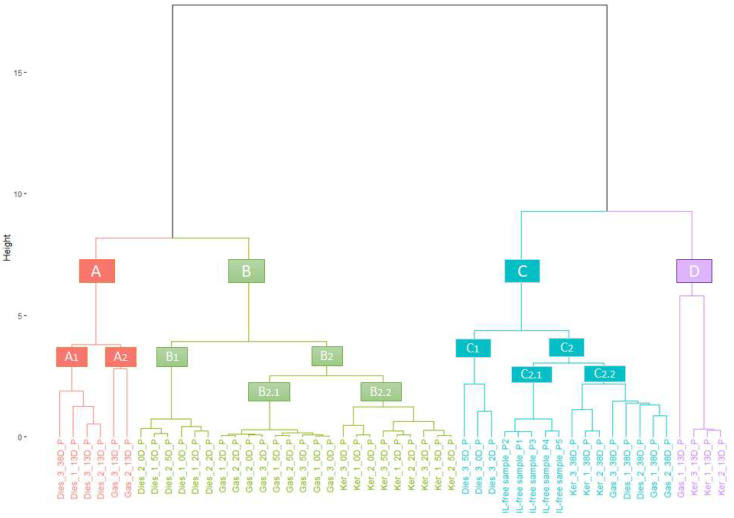
Dendrogram resulting from the analysis (M_50×990_).

The aim of this study was to investigate the potential of using IMSS for the characterization of different ILs biodegraded at different levels within soil samples (0, 2, 5, 13 and 38 days). Thus, the IMSS of all the biodegraded samples and IL-free samples were obtained (*n* = 100). First of all, in order to determine the biodegradation effect and how this phenomenon can interfere with the correct identification of the presence/absence and type of IL, an HCA was performed. For this analysis a reduced data matrix corresponding to the average of the replicates and the signal collected between 1020 and 2010 ms was used (M_50×990_). Wards method with Euclidean distance was chosen for the HCA. The results have been graphically displayed in the dendrogram in Figure 1.

The dendrogram shows a significant tendency of the samples to be grouped according to the type of IL as well as to the biodegradation time length. This trend is even clearer when the ILs have not been severely biodegraded. In fact, the majority of the samples that had been biodegraded for 0, 2 and 5 days are located in cluster B, where a subcluster (B_1_) can also be found containing just diesel oil samples. A second subcluster B_2_ can be identified, which is further divided into two additional subclusters; one populated by gasoline samples (B_2.1_) and the other one by kerosene samples (B_2.2_). The 13 day-biodegraded samples were grouped in either cluster A (which includes two different subgroups that correspond to diesel oil (A_1_) and gasoline samples (A_2_)) or cluster D, that groups the 13-D kerosene samples plus a particular exception: the Gas_1_13D_P sample, which was misclassified into this group.

Cluster C is divided into two subclusters, C_1_ and C_2_. It can also be observed that C_2_ includes two subclusters: C_2.1_ and C_2.2_, the former one contains all the soil samples that were free from ILs, while the latter grouped all the 38D most severely biodegraded samples. It seems evident that the samples tend to be grouped according to two different factors, i.e., their IL type and the degree of degradation. For samples biodegraded for short periods (2–8 days), clear groups were formed based on the IL. However, for samples biodegraded for long periods (up to 38 days), the groups of samples with the same IL were less homogeneous. Therefore, the higher degree of biodegradation, the higher the similarity among samples from containing different ILs. It should also be highlighted that subcluster C_1_ contains just the brand 3 diesel samples that had been degraded for 0, 2 and 5 days. This suggests that IMSS results are greatly influenced by this diesel brand specific characteristics.

To further analyze those diesel samples (brand “3” diesel oil), their Silhouette coefficients were calculated. Silhouette is an internal cluster validation method that quantifies how accurately cluster assignments have been performed in each case. The centroid is the average value in each group (Cluster A, B, C and D). As can be observed in Table 3, the silhouette coefficients of the samples in cluster C are high, except for those corresponding to brand 3 diesel oil samples after 0, 2 and 5-day of biodegradation. This indicates that, although they have been positioned within cluster C, their classification lacks the desired accuracy level. In fact, cluster B was the neighbor cluster for brand 3 diesel oil samples degraded for 0 and 2 days (similarly to the rest of the samples that had undergone biodegradation for the same time). The neighbor cluster for sample Dies_3_5D_P was A (which also includes sample “Dies_3_13D_P”).

A final cluster resampling test was carried out to evaluate their significance. The bootstrap-resampling value used was 10,000. The results are shown in Figure S1 (Supplementary Material), where we can see the *p*-value scores in red color: AU (Approximately Unbiased); BP (Bootstrap Probability). Most of the scores represent a very high probability, with values over 95%, which indicates that the clusters have not been indiscriminately defined.

Based on this, we can confirm that although HCA’s results did not achieve a precise separation of all the samples, they present a grouping trend that is based on IL type. This indicates that the data obtained from the IMSS are related to the compounds responsible for the discrimination of the different ILs, even if they are affected by the samples’ degradation level. Since the 990 signals corresponding to the samples’ drift times were not sufficient to achieve a full separation of the analyzed samples, an LDA that would allow the selection of the relevant IMSS data with regards to ILs presence and type, regardless of the samples’ biodegradation level, was carried out. Prior to performing the LDA, it was necessary to reduce the number of drift times. For this purpose, a data smoothing process was performed by selecting 3-drift time averages. The resulting reduced matrix (D_100×330_) was then employed for the LDA.

Four groups corresponding to each one of the three IL types (Gasoline, Diesel, Kerosene) and to the IL-free samples were previously established. The most significant drift times for the LDA were selected according to a supervised stepwise method. Wilke’ Lambda value was used as the criterion to include or remove the different variables. The input *F*-value was 3.84 and the output *F*-value was 2.71. A leaving-one-out cross-validation was carried out to assess the LDA performance.

A reduced number of 23 drift times were selected from the analysis to produce the discriminant functions: 1.021, 1.078, 1.095, 1.134, 1.151, 1.162, 1.168, 1.188, 1.196, 1.202, 1.225, 1319, 1.359, 1.409, 1.538, 1.541, 1.583, 1.643, 1.723, 1.747, 1.753, 1.787 and 1.906.

The canonical correlations of the discriminant functions were 0.997, 0.983, and 0.942 for F1, F2 and F3 respectively. The resulting functions allowed a 99% successful classification of the samples with the exception of sample “Gas_3_38D_R1”, which was misclassified as a kerosene sample. Since this is a 38-day biodegraded sample, it is not surprising that its degradation level makes of an accurate discrimination a really challenging objective.

**Figure 2:**
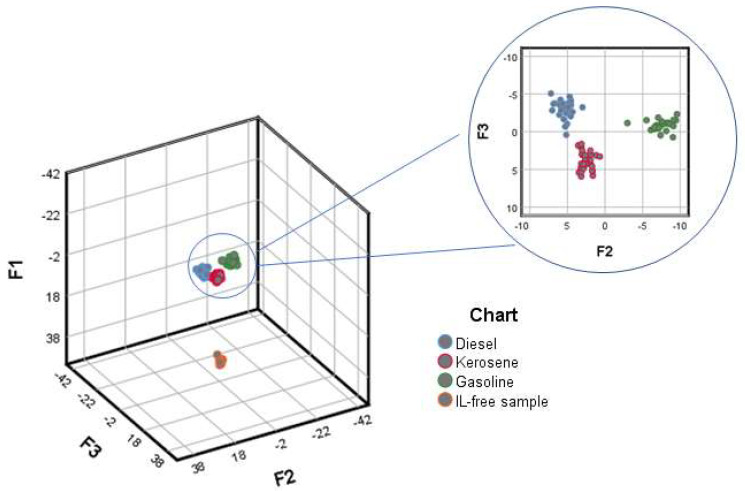
Discriminant scores obtained for all the samples (D_100×330_).

Figure 2 illustrates the distribution of the samples according to the three discrimination functions (F1, F2 and F3). It can be seen that F1 allows the space differentiation of IL-free samples (negative loading values) from ILs samples (positive loading values), which is essential to detect IL presence/absence. With regards to the discrimination between IL types, two groups are differentiated by F2, the first one corresponds to the gasoline samples (negative loading values) and the second one to the diesel oil and kerosene samples (positive loading values). F3 allows the discrimination of the soil samples that contain diesel oil (negative loading values) from those containing kerosene (positive loading values). Based on these results, the combination of F2 with F3, on the one hand, is the main reference for the discrimination of the samples according to IL type, while F1, on the other hand, is related to the detection of ILs in general. These results confirm the capacity of the method used to detect and classify ILs, even after they have been biodegraded for periods as long as 38 days.

With the purpose of developing a simple, fast and reliable method based on a fingerprint, the 2 drift times that presented the highest values in the standardized canonical discriminant function coefficient from each discriminant function were selected. Accordingly, 6 drift times were used (1.747, 1.753, 1.202, 1.319, 1.541 and 1.538).

**Figure 3:**
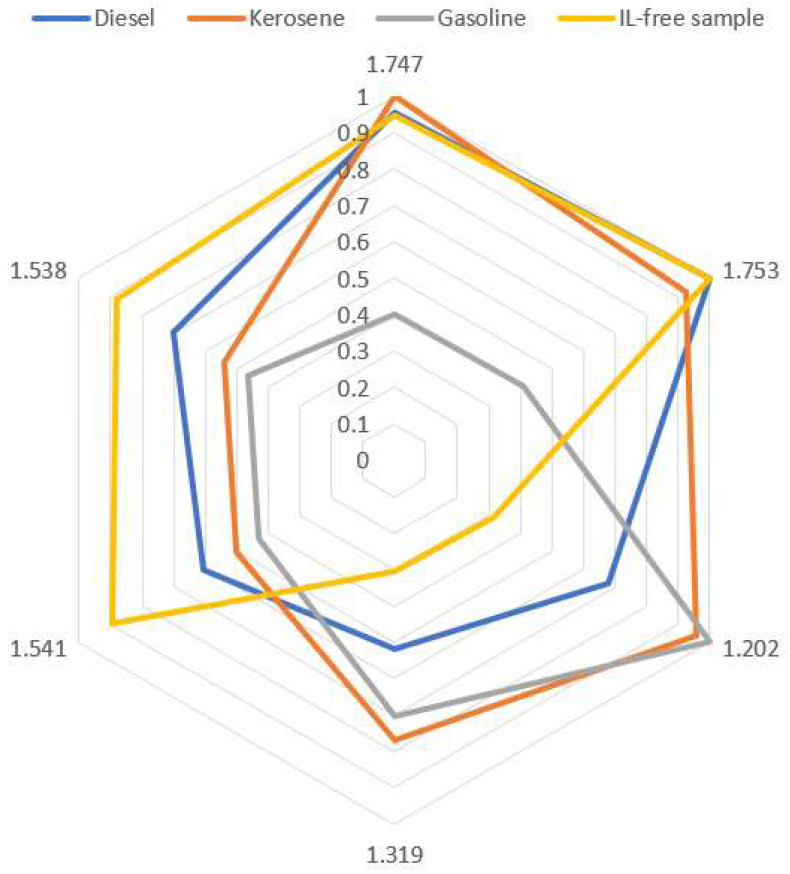
Fingerprint resulting from the drift times selected from the linear discriminant analysis (LDA).

The average intensities corresponding to each one of the sample categories (IL-free sample, soil with gasoline, with diesel oil and with kerosene) were calculated for each one of the 6 drift times that had been selected and normalized to each sample’s maximum signal. The results are shown in Figure 3. A characteristic fingerprint for each category was obtained. As can be seen, the soil that was free from any type of IL exhibited its maximum intensities at 1.753, 1.747, 1.538 and 1.541 drift time, while at 1.319 and 1.202 drift time, its intensities were below 0.5 (50% of its maximum value). This profile is completely different from the one corresponding to the samples containing some type of IL. In the particular case of gasoline samples, their maximum intensities were measured at 1.202 and 1.319 drift time, while their intensity values went below 0.5 at the rest of the drift times. The soil samples containing diesel oil or kerosene presented intensities above 0.5 at all the drift times. These profile similarities can be considered as logical, since they have similar chemical compositions. In addition, according to ASTM 1618, diesel oil and kerosene are found within the same IL category “heavy chain petroleum distillates” [1]. However, some differences can be observed, being more remarkable at 1.319 and 1.202 drift times. Such differences, as well as the ratios between the different signals should be considered as relevant, since they allow to determine the presence/absence as well as to identify the type of IL.

## 4. Conclusions

The suitability of IMSS in combination with pattern recognition techniques to determine the presence/absence as well as the IL types after different biodegradation times (from 0 to 38 days) has been evaluated. Microbial degradation processes have demonstrated to have a strong influence on the IMSS resulting from the headspace of soil samples containing gasoline, diesel oil or kerosene.

However, the implementation of an adequate pattern recognition technique such as LDA to the IMSS results has allowed 99% of the samples to be successfully discriminated.

Based on these results, we consider that the use of IMSS together with the suitable chemometric tools, and particularly LDA, represents a functional alternative to other methods often used for the detection and classification of ILs, even when the samples have been biodegraded for over a month time. In addition, a fingerprint based on 6 drift times has been elaborated for an automated database searching and a fast, objective and classification of the samples. Although a greater variety of ILs, substrates and biodegradation times should also be investigated, the results from this study suggest that IMSS presents an excellent potential for IL classification in fire investigations.

## Figures and Tables

**Table 1:** Properties of the different pretreatment techniques.

Technique/Property	ACS	Solid Phase Microextraction	Adsorption/Desorption Using Tenax	Direct Analysis
Cost	Moderate	High	Moderate	Low
Analysis time	Long	Short	Short	Short
Routine Application	Moderate	Moderate	Easy	Easy
Sensitivity	High	High	High	Moderate
Hazard	Moderate	None	None	None
Sample Handling	Large	None	Moderate	None
Risk of sample contamination	Moderate	High	Moderate	Low
Environmentally pollution	High	Low	Low	Very Low
Option to run duplicates on the same sample	Yes	No	No	No
Portability	Partially	Partially	No	Yes

**Table 2:** Conditions used for the analysis of samples in headspace–gas chromatography–ion mobility spectroscopy (HS-GC-IMS).

HS Conditions	Value
Incubation time (min)	15
Incubation temperature (°C)	50
Agitation speed (rpm)	750
Injection volume (µL)	250
Syringe filling speed (µL/s)	850
Syringe temperature (°C)	55
Flushing time (min)	5
Injection speed (µL/s)	850
**GC-IMS Conditions**	**Value**
Electronic pressure control 1 (mL/min)	250
Electronic pressure control 2 (mL/min)	2 mL/min (t = 0 min); 10 mL/min (t = 5 min); 25 mL/min (t = 10 min)
IMS temperature (°C)	45
Column temperature (°C)	55
Total analysis time (min)	25

**Table 3:** Silhouette coefficients for cluster C.

Samples	Real Cluster	Neighbor	Silhouette Coefficient
IL-free sample_P1	C	A	0.6632
IL-free sample_P3	C	A	0.6629
IL-free sample_P4	C	A	0.6585
IL-free sample_P5	C	A	0.6576
IL-free sample_P2	C	A	0.6543
Gas_2_38D_P	C	A	0.6450
Gas_1_38D_P	C	A	0.6133
Ker_2_38D_P	C	D	0.6099
Ker_3_38D_P	C	A	0.5991
Dies_2_38D_P	C	D	0.5895
Ker_1_38D_P	C	D	0.5840
Dies_1_38D_P	C	A	0.5543
Gas_3_38D_P	C	A	0.5163
Dies_3_2D_P	C	B	0.3854
Dies_3_0D_P	C	B	0.3148
Dies_3_5D_P	C	A	0.0572

## Supplementary Materials

The following are available online at https://www.mdpi.com/1424-8220/20/21/6005/s1, Figure S1: Dendrogram resulting from the analysis (M_50×990_). Values at branches are AU *p*-values (left), BP values (right), and cluster labels (bottom). Clusters with AU ≥ 95 are indicated by the rectangles.
