# Supplementary material for: Characterization of Biodegraded Ignitable Liquids by Headspace–Ion Mobility Spectrometry
Source: Sensors (Basel). 2020 Oct 23;20(21):6005. doi: 10.3390/s20216005 (PMC7660173; doi:10.3390/s20216005)
Supplement: Supplementary file 1 [file sensors-20-06005-s001.pdf]

Characterization of Biodegraded Ignitable Liquids by Headspace - Ion Mobility Spectrometry

José Luis P. Calle, Marta Ferreiro-González *, María José Aliaño-González, Gerardo F. Barbero and Miguel Palma

Department of Analytical Chemistry, Faculty of Sciences, Agrifood Campus of International Excellence (ceiA3), IVAGRO, University of Cadiz, 11510 Puerto Real, Cadiz, Spain; joseluis.perezcalle@uca.es (J.L.P.C.); marta.ferreiro@uca.es (M.F.-G.); mariajose.alianogonzalez@alum.uca.es (M.J.A.-G.); gerardo.fernandez@uca.es (G.F.B.); miguel.palma@uca.es (M.P.)

* Correspondence: marta.ferreiro@uca.es (M.F-G.); Tel.: +34-956016359

SUPPLEMENTARY MATERIAL

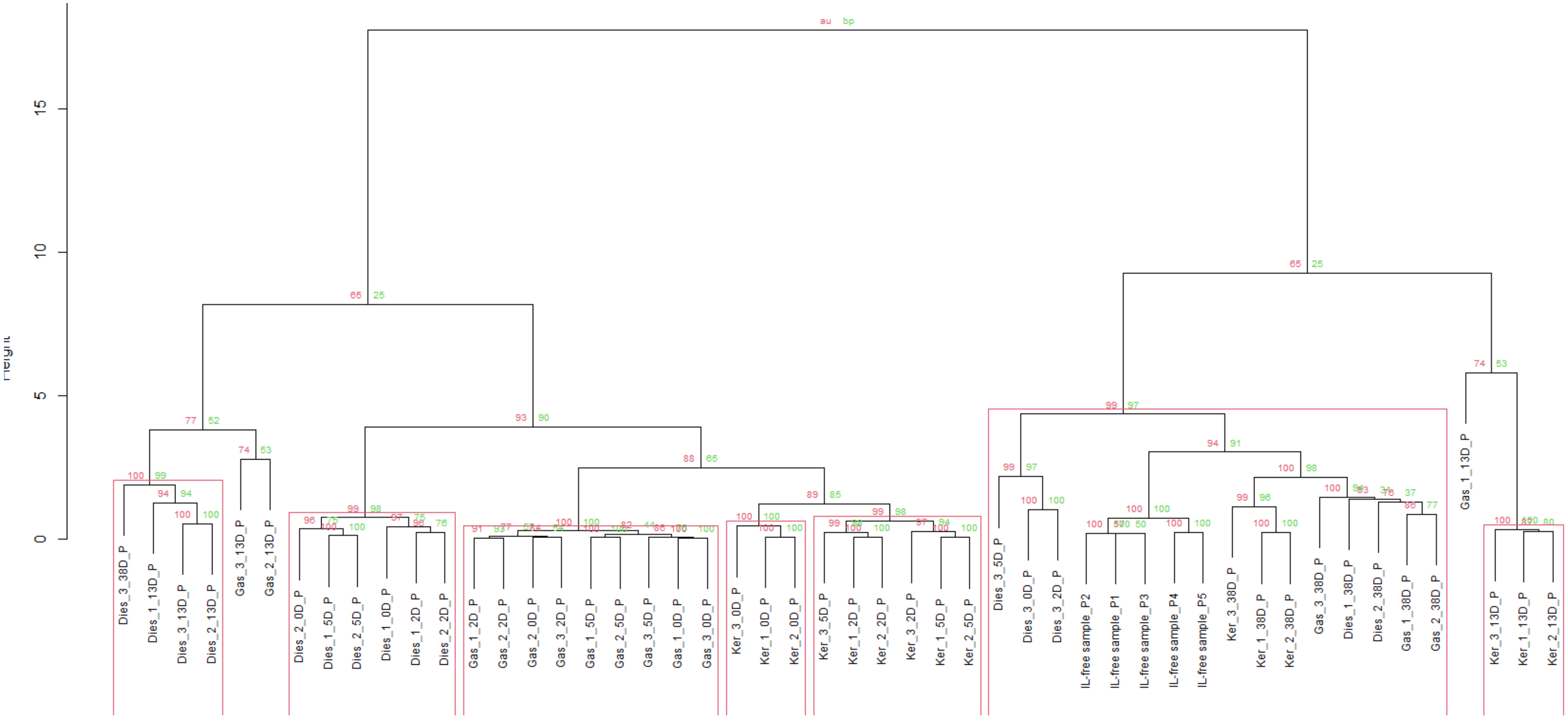

Figure S1. Dendrogram resulting from the analysis (M_{50x990}). Values at branches are AU *p*-values (left), BP values (right), and cluster labels (bottom). Clusters with AU ≥ 95 are indicated by the rectangles.
